# 1057. Tebipenem *In vitro* Activity Against a Collection of Pathogens Responsible for Urinary Tract Infections in the US

**DOI:** 10.1093/ofid/ofab466.1251

**Published:** 2021-12-04

**Authors:** Rodrigo E Mendes, Ian A Critchley, Nicole Cotroneo, Jennifer M Streit, Helio S Sader, Mariana Castanheira, Mariana Castanheira

**Affiliations:** 1 JMI Laboratories, North Liberty, Iowa; 2 Spero Therapeutics, Cambridge, Massachusetts

## Abstract

**Background:**

*Enterobacterales* (ENT)—especially *Escherichia coli* (EC), *Klebsiella pneumoniae* (KPN), and *Proteus mirabilis* (PM)—are widely implicated in urinary tract infections (UTIs). Many oral agents are used to manage UTIs, but their usefulness has been compromised by the increased prevalence of extended-spectrum β-lactamases (ESBL) and presence of co-resistance to trimethoprim-sulfamethoxazole (TMP/SMX) and quinolones. Tebipenem (TBP) is an oral carbapenem in clinical development for treating complicated UTIs and acute pyelonephritis. This study assessed the *in vitro* activity of TBP and comparator agents against ENT responsible for UTIs in the US during 2019-2020.

**Methods:**

A total of 3,576 ENT recovered from urine samples during the 2019-2020 STEWARD Surveillance Program were included in the study. Isolates were collected from medical centers in all 9 US Census Regions and were centrally tested for susceptibility by reference broth microdilution method. MIC interpretation was performed based on CLSI criteria.

**Results:**

EC comprised 65.6% of all ENT pathogens, followed by KPN (14.3%), PM (6.6%), and other species (13.7%). TBP (MIC_90_, 0.015-0.06 mg/L) and ertapenem (ERT; MIC_90_, 0.03 mg/L) showed similar MIC_90_ results against ENT, EC, and KPN (Table). Ceftazidime (CAZ; MIC_90_, 8-16 mg/L) had elevated MIC_90_ values and suboptimal susceptibility results (86.1-89.3%) against ENT, EC, and KPN. The oral agents, cefuroxime, amoxicillin-clavulanate, TMP-SMX, and levofloxacin showed susceptibility rates ranging from 63.1% to 87.1% against ENT, EC, and KPN. TBP (MIC_50/90_, 0.12/0.12 mg/L) inhibited all PM at ≤0.25 mg/L. PM isolates were susceptible to ERT (100.0%), CAZ (98.7%), cefuroxime (94.4%), and amoxicillin/clavulanate (96.6%), whereas susceptibility rates of 71.8-76.8% were noted for TMP-SMX and levofloxacin.

**Conclusion:**

TBP displayed potent activity against ENT UTI pathogens recovered from patients in the US. TBP demonstrated *in vitro* activity against these UTI pathogens similar to that of ERT. In addition, these data showed compromised activity of intravenous and oral agents used for treating UTI. This data supports the development of tebipenem as an oral option for management of UTI in the US.

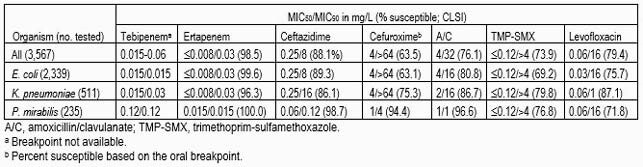

**Disclosures:**

**Rodrigo E. Mendes, PhD**, **AbbVie** (Research Grant or Support)**AbbVie (formerly Allergan**) (Research Grant or Support)**Cipla Therapeutics** (Research Grant or Support)**Cipla USA Inc.** (Research Grant or Support)**ContraFect Corporation** (Research Grant or Support)**GlaxoSmithKline, LLC** (Research Grant or Support)**Melinta Therapeutics, Inc.** (Research Grant or Support)**Melinta Therapeutics, LLC** (Research Grant or Support)**Nabriva Therapeutics** (Research Grant or Support)**Pfizer, Inc.** (Research Grant or Support)**Shionogi** (Research Grant or Support)**Spero Therapeutics** (Research Grant or Support) **Ian A. Critchley, Ph.D.**, **Spero Therapeutics** (Employee, Shareholder) **Nicole Cotroneo**, **Spero Therapeutics** (Employee, Shareholder) **Jennifer M. Streit, BS**, **GlaxoSmithKline, LLC** (Research Grant or Support)**Melinta Therapeutics, LLC** (Research Grant or Support)**Shionogi** (Research Grant or Support)**Spero Therapeutics** (Research Grant or Support) **Helio S. Sader, MD, PhD, FIDSA**, **AbbVie (formerly Allergan**) (Research Grant or Support)**Basilea Pharmaceutica International, Ltd.** (Research Grant or Support)**Cipla Therapeutics** (Research Grant or Support)**Cipla USA Inc.** (Research Grant or Support)**Department of Health and Human Services** (Research Grant or Support, Contract no. HHSO100201600002C)**Melinta Therapeutics, LLC** (Research Grant or Support)**Nabriva Therapeutics** (Research Grant or Support)**Pfizer, Inc.** (Research Grant or Support)**Shionogi** (Research Grant or Support)**Spero Therapeutics** (Research Grant or Support) **Mariana Castanheira, PhD**, **AbbVie (formerly Allergan**) (Research Grant or Support)**Bravos Biosciences** (Research Grant or Support)**Cidara Therapeutics, Inc.** (Research Grant or Support)**Cipla Therapeutics** (Research Grant or Support)**Cipla USA Inc.** (Research Grant or Support)**GlaxoSmithKline** (Research Grant or Support)**Melinta Therapeutics, Inc.** (Research Grant or Support)**Melinta Therapeutics, LLC** (Research Grant or Support)**Pfizer, Inc.** (Research Grant or Support)**Qpex Biopharma** (Research Grant or Support)**Shionogi** (Research Grant or Support)**Spero Therapeutics** (Research Grant or Support) **Mariana Castanheira, PhD**, Affinity Biosensors (Individual(s) Involved: Self): Research Grant or Support; Allergan (Individual(s) Involved: Self): Research Grant or Support; Amicrobe, Inc (Individual(s) Involved: Self): Research Grant or Support; Amplyx Pharma (Individual(s) Involved: Self): Research Grant or Support; Artugen Therapeutics USA, Inc. (Individual(s) Involved: Self): Research Grant or Support; Astellas (Individual(s) Involved: Self): Research Grant or Support; Basilea (Individual(s) Involved: Self): Research Grant or Support; Beth Israel Deaconess Medical Center (Individual(s) Involved: Self): Research Grant or Support; BIDMC (Individual(s) Involved: Self): Research Grant or Support; bioMerieux Inc. (Individual(s) Involved: Self): Research Grant or Support; BioVersys Ag (Individual(s) Involved: Self): Research Grant or Support; Bugworks (Individual(s) Involved: Self): Research Grant or Support; Cidara (Individual(s) Involved: Self): Research Grant or Support; Cipla (Individual(s) Involved: Self): Research Grant or Support; Contrafect (Individual(s) Involved: Self): Research Grant or Support; Cormedix (Individual(s) Involved: Self): Research Grant or Support; Crestone, Inc. (Individual(s) Involved: Self): Research Grant or Support; Curza (Individual(s) Involved: Self): Research Grant or Support; CXC7 (Individual(s) Involved: Self): Research Grant or Support; Entasis (Individual(s) Involved: Self): Research Grant or Support; Fedora Pharmaceutical (Individual(s) Involved: Self): Research Grant or Support; Fimbrion Therapeutics (Individual(s) Involved: Self): Research Grant or Support; Fox Chase (Individual(s) Involved: Self): Research Grant or Support; GlaxoSmithKline (Individual(s) Involved: Self): Research Grant or Support; Guardian Therapeutics (Individual(s) Involved: Self): Research Grant or Support; Hardy Diagnostics (Individual(s) Involved: Self): Research Grant or Support; IHMA (Individual(s) Involved: Self): Research Grant or Support; Janssen Research & Development (Individual(s) Involved: Self): Research Grant or Support; Johnson & Johnson (Individual(s) Involved: Self): Research Grant or Support; Kaleido Biosceinces (Individual(s) Involved: Self): Research Grant or Support; KBP Biosciences (Individual(s) Involved: Self): Research Grant or Support; Luminex (Individual(s) Involved: Self): Research Grant or Support; Matrivax (Individual(s) Involved: Self): Research Grant or Support; Mayo Clinic (Individual(s) Involved: Self): Research Grant or Support; Medpace (Individual(s) Involved: Self): Research Grant or Support; Meiji Seika Pharma Co., Ltd. (Individual(s) Involved: Self): Research Grant or Support; Melinta (Individual(s) Involved: Self): Research Grant or Support; Menarini (Individual(s) Involved: Self): Research Grant or Support; Merck (Individual(s) Involved: Self): Research Grant or Support; Meridian Bioscience Inc. (Individual(s) Involved: Self): Research Grant or Support; Micromyx (Individual(s) Involved: Self): Research Grant or Support; MicuRx (Individual(s) Involved: Self): Research Grant or Support; N8 Medical (Individual(s) Involved: Self): Research Grant or Support; Nabriva (Individual(s) Involved: Self): Research Grant or Support; National Institutes of Health (Individual(s) Involved: Self): Research Grant or Support; National University of Singapore (Individual(s) Involved: Self): Research Grant or Support; North Bristol NHS Trust (Individual(s) Involved: Self): Research Grant or Support; Novome Biotechnologies (Individual(s) Involved: Self): Research Grant or Support; Paratek (Individual(s) Involved: Self): Research Grant or Support; Pfizer (Individual(s) Involved: Self): Research Grant or Support; Prokaryotics Inc. (Individual(s) Involved: Self): Research Grant or Support; QPEX Biopharma (Individual(s) Involved: Self): Research Grant or Support; Rhode Island Hospital (Individual(s) Involved: Self): Research Grant or Support; RIHML (Individual(s) Involved: Self): Research Grant or Support; Roche (Individual(s) Involved: Self): Research Grant or Support; Roivant (Individual(s) Involved: Self): Research Grant or Support; Salvat (Individual(s) Involved: Self): Research Grant or Support; Scynexis (Individual(s) Involved: Self): Research Grant or Support; SeLux Diagnostics (Individual(s) Involved: Self): Research Grant or Support; Shionogi (Individual(s) Involved: Self): Research Grant or Support; Specific Diagnostics (Individual(s) Involved: Self): Research Grant or Support; Spero (Individual(s) Involved: Self): Research Grant or Support; SuperTrans Medical LT (Individual(s) Involved: Self): Research Grant or Support; T2 Biosystems (Individual(s) Involved: Self): Research Grant or Support; The University of Queensland (Individual(s) Involved: Self): Research Grant or Support; Thermo Fisher Scientific (Individual(s) Involved: Self): Research Grant or Support; Tufts Medical Center (Individual(s) Involved: Self): Research Grant or Support; Universite de Sherbrooke (Individual(s) Involved: Self): Research Grant or Support; University of Iowa (Individual(s) Involved: Self): Research Grant or Support; University of Iowa Hospitals and Clinics (Individual(s) Involved: Self): Research Grant or Support; University of Wisconsin (Individual(s) Involved: Self): Research Grant or Support; UNT System College of Pharmacy (Individual(s) Involved: Self): Research Grant or Support; URMC (Individual(s) Involved: Self): Research Grant or Support; UT Southwestern (Individual(s) Involved: Self): Research Grant or Support; VenatoRx (Individual(s) Involved: Self): Research Grant or Support; Viosera Therapeutics (Individual(s) Involved: Self): Research Grant or Support; Wayne State University (Individual(s) Involved: Self): Research Grant or Support

